# A case report of COVID-19 monitoring in the Austrian professional football league

**DOI:** 10.1038/s41598-021-03820-9

**Published:** 2021-12-24

**Authors:** Antje van der Zee-Neuen, Dagmar Schaffler-Schaden, Jürgen Herfert, James O´Brien, Tim Johansson, Patrick Kutschar, Alexander Seymer, Stephan Ludwig, Thomas Stöggl, David Keeley, Herbert Resch, Jürgen Osterbrink, Maria Flamm

**Affiliations:** 1 Paracelsus Medical University, Institute of Nursing Science and Practice, Centre for Public Health and Health Services Research, Strubergasse 21, 5020 Salzburg, Austria; 2 Institute of General Practice, Family Medicine and Preventive Medicine, Paracelsus Medical University, Centre for Public Health and Health Services Research, Strubergasse 21, 5020 Salzburg, Austria; 3 Red Bull Athlete Performance Centre, Brunnbachweg 71, 5303 Thalgau, Austria; 4 The Australian Centre for Research Into Injury in Sport and Its Prevention (ACRISP), Edith Cowan University, 270 Joondalup Drive , Joondalup, WA 6027 Australia; 5 Department of Political Science and Sociology, University of Salzburg, Rudolfskai 42, 5020 Salzburg, Austria; 6 Institute of Virology (IVM), University of Muenster (WWU), Von-Esmarch-Straße 56, 48149 Muenster, Germany; 7 Department of Sport and Exercise Science, University of Salzburg, Schlossallee 49, 5400 Hallein, Austria; 8 Electronic Caregiver, Divison of Innovation, 506 S Main St STE. 1000, Las Cruces, NM 88001 USA; 9 University of North Florida, Brooks College of Health, Building 39, 1 UNF Drive, Jacksonville, FL 32224 USA

**Keywords:** Medical research, Epidemiology

## Abstract

Since the beginning of the COVID -19 pandemic, many contact sport teams are facing major challenges to safely continue training and competition. We present the design and implementation of a structured monitoring concept for the Austrian national football league. 146 professional players from five clubs of the professional Austrian football league were monitored for a period of 12 weeks. Subjective health parameters, PCR- test results and data obtained from a geo-tracking app were collected. Simulations modelling the consequences of a COVID-19 case with increasing reproduction number were computed. No COVID -19 infection occurred during the observation period in the players. Infections in the nearer surroundings lead to increased perceived risk of infection. Geo tracking was particularly hindered due to technical problems and reluctance of users. Simulation models suggested a hypothetical shut-down of all training and competition activities. A structured monitoring concept can help to continue contact sports safely in times of a pandemic. Cooperation of all involved is essential.

Trial registration: ID: DRKS00022166 15/6/2020 https://www.who.int/ictrp/search/en/.

## Introduction

Since the global outbreak of the COVID-19 pandemic, team contact sports are facing major challenges to continue training and competing. Medical recommendations to prevent virus transmission are changing on a day-to-day basis and although physical distancing is currently a widely accepted preventive measure, its application is not feasible in team contact sports^1^. Shared training facilities and close, prolonged contact with opponents and team members can increase transmission risk in these settings.

After a period of home-confined training, professional football teams all around the world have resumed training and competition. Since October 2021, Austria is experiencing a renewed and steep increase in cases, but fortunately the intensive care capacities are not exhausted^2^. Following a stabilisation in infection rates, many European countries eased strict measures to prevent COVID-19 transmission in summer 2020, including resuming public activities, reopening borders and increasing the accessibility of public places. Nevertheless, the upcoming winter and increased travel to international games pose new complex challenges for professional sport organisations^3^.

Recent data suggest that one-fourth of infected people show no symptoms, which hampers timely identification and adversely impacts the risk of unnoticed transmission^4–6^. As young and fit athletes may well be asymptomatic in the case of an infection, strict management in team contact sports context is warranted. In order to minimise transmission risk and limit potential strain on health services, the resumption of team sports necessitates a clearly defined, well-coordinated prevention strategy. Several concepts for safely resuming national and international competition have been published. These concepts reinforce the necessity of prevention strategies, highlight the importance of days between matches and the frequency of virus tests on the number of players infected and suggest that the limitation of personal contact and travel movement would be most ideal in preventing infection among players. Moreover, the relevance of a controlled environment is reinforced in which the safety of athletes and those in their environment should be prioritised and promoted^7–9^.

On May 12th 2020, the Austrian government granted permission for the resumption of the Austrian national male association football league, on the condition of no spectators and teams adhering to a pre-defined prevention concept. A further condition was scientific monitoring of the resumption process. The authors were involved in designing and implementing this monitoring concept in selected teams. The overarching goal of the prevention concept and scientific evaluation was to allow safe resumption of training and competition through comprehensive health monitoring and tracking of players’ movements both in- and outside the trainings and competition facilities.

The current manuscript reports the design of the monitoring concept, the barriers encountered during the implementation process and also presents simulation models for infections. In addition to describing the monitoring parameters and their variation across the observation period, a statistical estimation of consequences of a rise in COVID-19 basic reproduction number (R(0)) is provided. The R(0) is an indicator of the number of people infected by an infected person^10^. We simulated the transmission and spread of the virus in a football team when the R(0) rises (e.g. R(0) > 1). The design and implementation of the monitoring concept, including the lessons learned can serve as a valuable blueprint for the safe resumption of professional, recreational and educational sports during a pandemic.

## Methods

The monitoring concept was evaluated across a 12-week period from May 15th 2020 to the end of the football season on July 5th 2020. Players from five consenting Austrian professional football clubs (highest two divisions) were included. For reasons of anonymity, clubs were given code-names (A-E).

### Instruments and measures

For the monitoring of health and movement outside training/game facilities, the instruments and their content were structured in three categories:

#### Health diaries

Diaries included several single questions relating to potential COVID-19 symptoms:

- Cough during the last 24 h (yes/no)
- Breathing difficulties during the last 24 h (yes/no)
- Loss of sense of smell/taste during the last 24 h (yes/no)
- Body aches during the last 24 h (yes/no)
- Proxies for anxiety, namely
- Sleep quality (10-point scale, 1 = bad sleep, problems falling asleep, several night time wake-ups, 10 = good sleep, quickly fell asleep slept through the night)
- Current perceived risk of infection (10-point scale, 1 = calm, composed, unconcerned 10 = panicked, anxious, severe risk) as a proxy for anxiety related to the pandemic
- Current perceived recovery state (10-point scale, 1 = energetic, cheerful, rested 10 = tired, unenthusiastic, exhausted)

#### Objective parameters

- COVID-19 real time polymerase chain reaction (RT-PCR) tests (by means of nasal and/or throat swab)—screening took place once per week and pooled testing was permitted (i.e. in case of positives in one pool, individual tests must be performed). Analyses of samples were performed by laboratories as recommended by the Federal Ministry of the Republic of Austria, Social Affairs, Health, Care and Consumer Protection
- Body temperature (in Celsius)—measured daily by means of a contactless, forehead thermometer
- Oxygen saturation (SpO2)—measured daily by means of a pulse oximeter attached to the left index finger
- Geo-tracking data:
  Geo-tracking data allowed monitoring of players’ movement outside the training/competition facilities. This digital parameter was measured using a smartphone application (app), which was specifically designed by the US-based company Electronic Caregiver for the purpose of data collection and installed on players’ primary mobile devices. This app collected motion data triggered by Global Navigation Satellite System (GNSS) data points. The data was stored in flexible schemes of a noSQL database (i.e. data was stored and retrieved without predefined structure), in order to gather the multiple layers of information capture associated with each data point.

Table 1 provides an overview of all parameters, including the mode and frequency of measurement and the individuals collecting the data.

**Table 1:** Overview of included parameters.

Parameter	Mode of measurements	Frequency of measurement	Rater/data-collector
**Health diaries**			
Sleep quality	Single question, self-perceived rating	Before training or competition	Team physiotherapist
Level of recovery	Single question, self-perceived rating	Before training or competition	Team physiotherapist
Perceived risk of infection	Single question, self-perceived rating	Before training or competition	Team physiotherapist
Loss of sense of smell/taste	Single question, self-perceived rating	Before training or competition	Team physiotherapist
Body aches	Single question, self-perceived rating	Before training or competition	Team physiotherapist
Cough	Single question, self-perceived rating	Before training or competition	Team physiotherapist
Difficulties in breathing	Single question, self-perceived rating	Before training or competition	Team physiotherapist
**Objective parameters**			
COVID-19 RT-PCR	Throat or nasal swab	Weekly	Team physician
Body temperature	Contactless, forehead	Before training or competition	Team physiotherapist
Oxygen saturation (SpO2)	Pulse oximeter (finger)	Before training or competition	Team physiotherapist
**Geo tracking**			
Geo-tracking data	Smartphone	Continuous	Mobile Application

### Data collection procedures and data quality assurance

During the observation period, standardized data collection sheets were distributed to participating clubs on a weekly basis. Data were collected by team physicians (i.e. COVID-19 RT-PCR tests) and physiotherapists, henceforth referred to as club-internal raters.

A contact person from the Paracelsus Medical University Salzburg (PMU) sent weekly reminders and updates to participating clubs. Three external, student raters, were employed and trained by the researchers to assist the club-internal raters with the collection of health data and the installation of the smartphone app for geo-tracking. The researchers cleaned the data and established a database in preparation, for statistical analysis using R version 3.5.2, R Core Team, 2018.

### Statistical analyses and presentation of results

#### Health diary data and objective parameters

For the purpose of the current manuscript, descriptive statistics were employed according to parameters metric properties: Mean and standard deviation (SD) or, after evaluation of distribution normality, median and interquartile range (IQR) for continuous variables and the total number (*n*) and percentage (%) for categorical variables.

In club A, a management employee, who had no direct contact with the players, tested positive to COVID-19 during the study period. To compare the subjective perceived risk of infection in players from club A before and after this confirmed COVID-19 case, a Wilcoxon-signed-rank test with continuity correction was performed accounting for data skewness. The test compared the mean score of infection risk per player one week prior to the COVID-19 case to the mean score of infection risk one week after the case.

#### Geo-tracking

To determine whether the range of movement of all included players varied substantially from their expected movements (i.e. in the proximity of their home and training and competition facilities) heat maps were generated from the geo-tracking data.

#### Simulation of COVID-19 case and potential consequences according to reproduction number R(0)

The impact of a hypothetical COVID-19 case on the continuation of training and competition in a participating club were simulated for randomly picked R(0)s. For this purpose, 30 players (as a nearly closed cohort) were considered and an observation period of 90 days was assumed. The simulation made the following assumptions: (1) the development of symptoms takes (approximately) 5 days, (2) players are tested every 5 days, (3) positively tested players are sent into a quarantine of 14 days, (4) all players had close contact and an identical risk to be infected by their teammates, (5)the number of new infected players depends on the R(0) of the spreading player, (6) newly infected players may be identified at the subsequent RT-PCR-test, (7) after players return from their quarantine, they do not get infected with COVID-19 again, (8) on day 1 all players are negative and on day 2 a randomly picked player gets infected with COVID-19, (9) infections are spread uniquely to a single teammate (i.e. two infected players cannot spread COVID-19 to the same teammate).

As an example, we considered simulations for population reproduction rates of R(0) = 1.4 and R(0) = 2.15. First, a vector of different R(0) simulated a normal distribution with the mean 1.4 and 2.15, respectively. The distributions had standard deviations of half the mean and a minimum of 0 and a maximum of two times the mean. To account for variation in the number of persons infected by one infected individual, a R(0) was drawn randomly from the vector of R(0) for every player. Hence, each player had a unique R(0) representing the average R(0) of the player over all 90 days. For the number of infections of teammates on a specific day, only the integer number of the R(0) was considered and the digits were added to the next day’s R(0). For example, if a player was positive on day 2 and had an R(0) = 1.6, the player infected one player on day 2. The R(0) for day 3 was 2.2 (1.6 + 0.6), meaning on day 3 the player infected two teammates. On day 4, the player had a R(0) of 1.8 (1.6 + 0.2) infecting one teammate. The simulation started without any infection on day 1. On day 2, a random player got infected starting to spread COVID-19 among their teammates on day 3. In order to consider different severities in the course of the spread of the virus, simulations were repeated 100 times for R(0) = 1.4 and for R(0) = 2.15, respectively.

#### Encountered implementation barriers.

Barriers to implementing the monitoring concept, as encountered by the researchers during the study period, were documented and summarized descriptively.

### Ethics

The study protocol and all procedures reported in this manuscript were approved by the Austrian ethics committee of Salzburg county (statement of the ethics board of Salzburg county, ID 415-EP/73/820–2020).

### Informed consent

Informed consent was obtained from all individual participants involved in the study. Participants were informed about the study purpose and procedures in writing and were additionally informed in person prior to providing their written consent.

### Guidelines and regulations

All procedures performed in the study were in accordance with the ethical standards of the Austrian ethics committee of Salzburg county and with the 1964 Helsinki Declaration and its later amendments.

## Results

The full sample consisted of 146 players from five clubs (club A, *n* = 27; club B, *n* = 30; club C, *n* = 28; club D, *n* = 29, club E, *n* = 32).

### Health diary data and objective parameters

**Figure 1:**
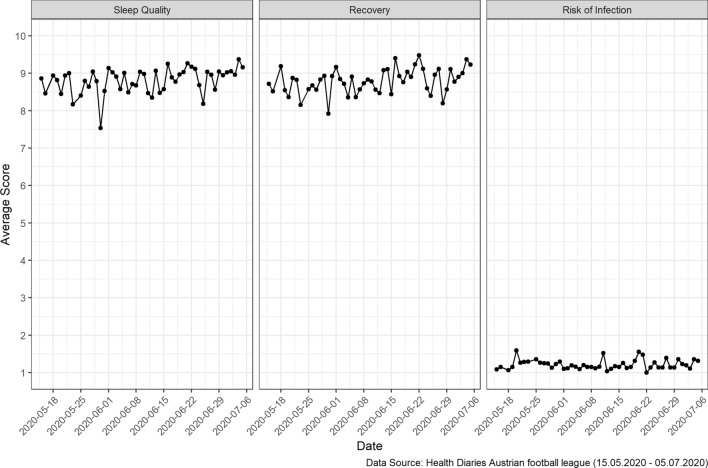
Mean score of perceived sleep quality, level of recovery and risk of infection over time across all participating players. (scales 1–10: 1-bad sleep-10-good sleep; recovery: 1-energetic – 10-tired; risk of infection: 1-calm – 10 anxious.

Only 4 (14.29%) club C players and 1 (3.45%) club D player reported a sore throat, 2 (7.14%) club C players reported coughing and 1 (3.57%) club C player reported body aches. Health parameters per club are presented in Table 2. The reported and measured health parameters suggest that players from all clubs were in good health during the observation period. None of the players tested positive for COVID-19. However, some variation was found in the parameters sleep quality, level of recovery and perceived risk of infection (Fig. 1). In club A, the players’ perceived risk of infection in the week after a manager tested positive for COVID-19 was significantly higher than the perceived risk in the week prior to the occurrence (V = 25; *p* < 0.001; effect-size = 0.73).

**Table 2:** Descriptive statistics of collected health parameters according to club over the entire observation period.

	Football clubs				
	Club A *n* = 1091*	Club B *n* = 1277*	Club C *n* = 1173*	Club E *n* = 675*	Club F *n* = 912*
**Body temperature [**°**C]**					
Mean (SD)	36.33 (0.26)	36.52 (0.21)	35.86 (0.33)	36.39 (0.35)	36.30 (0.35)
Oxygen saturation [%]	*N* (*valid*) = 365	*n*(*valid*) = 1009	*n*(*valid*) = 227	*n*(*valid*) = 297	*n*(*valid*) = 351
Mean (SD)	98.45 (0.67)	98.32 (0.76)	98.21 (0.69)	97.77 (1.05)	97.86 (1.02)
**Sleep quality (1–10)**					
Median (IQR)	2 (2–3)	1 (1–1)	2 (2–3)	1 (1–1)	2 (2–3)
**Recovery (1–10)**					
Median (IQR)	2 (2–3)	1 (1–1)	2 (2–4)	1 (1–1)	2 (2–3)
**Risk of infection (1–10)**					
Median (IQR)	1 (1–2)	1 (1–1)	1 (1–1)	1 (1–1)	1 (1–1)
Sore throat, yes, n** (%)	0 (0)	0 (0)	15 (1.28)	1 (0.15)	0 (0)
Cough, yes, n** (%)	0 (0)	0 (0)	10 (0.85)	0 (0)	0 (0)
Body aches, yes, n** (%)	0 (0)	0 (0)	1 (0.09)	0 (0)	0 (0)
Breathing difficult, yes, n** (%)	0 (0)	0 (0)	1 (0.09)	0 (0)	0 (0)
Loss of taste, yes, n** (%)	0 (0)	0 (0)	0 (0)	0 (0)	0 (0)
COVID-19 infection, yes n (%)	0 (0)	0 (0)	0 (0)	0 (0)	0 (0)

*Denotes the number of data points during the observation period by club.

**Denotes the number of times a specific symptom was reported (during the whole observation period).

*IQR* Interquartile range (1st quartile and 3rd quartile), *SD* standard deviation.

### Geo-tracking

**Figure 2:**
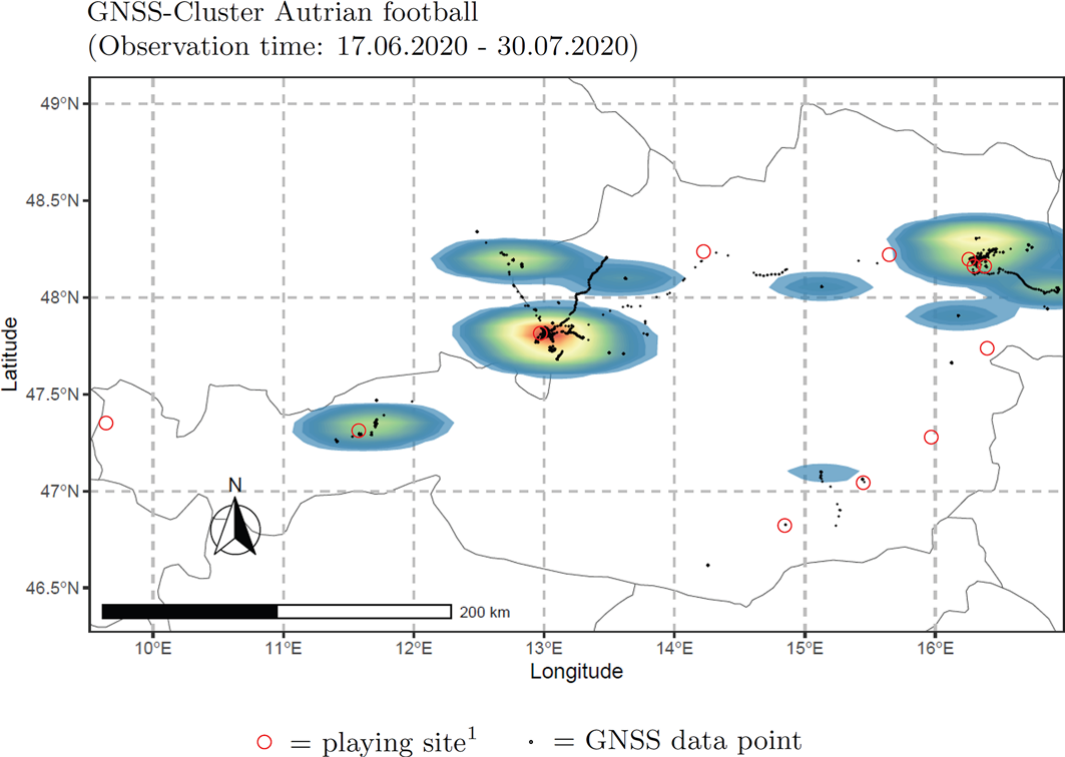
Heat map of collected geo tracking data of participants of five clubs of the Austrian Bundesliga (time period: June 17th 2020–July 30th 2020). The color gradient can be interpreted as follows: areas with high movement frequencies are displayed with red, areas with less movement frequencies are colored with blue. Heat map created using R version 3.5.2, R Core Team, 2018 (https://www.r-project.org/); country map drawn with package ‘maps’, original S code by Richard A. Becker, Allan R. Wilks. R version by Ray Brownrigg. Enhancements by Thomas P Minka and Alex Deckmyn. (2018). maps: Draw Geographical Maps. R package version 3.3.0 (reusable under GPL-2 License).

The collected geo-tracking data suggest that participants’ geographical movement was largely restricted to areas in the proximity of their training facilities and football stadiums they travelled to for the purpose of external matches (Fig. 2).

### Simulation of COVID-19 case and potential consequences according to reproduction number R(0)

**Figure 3:**
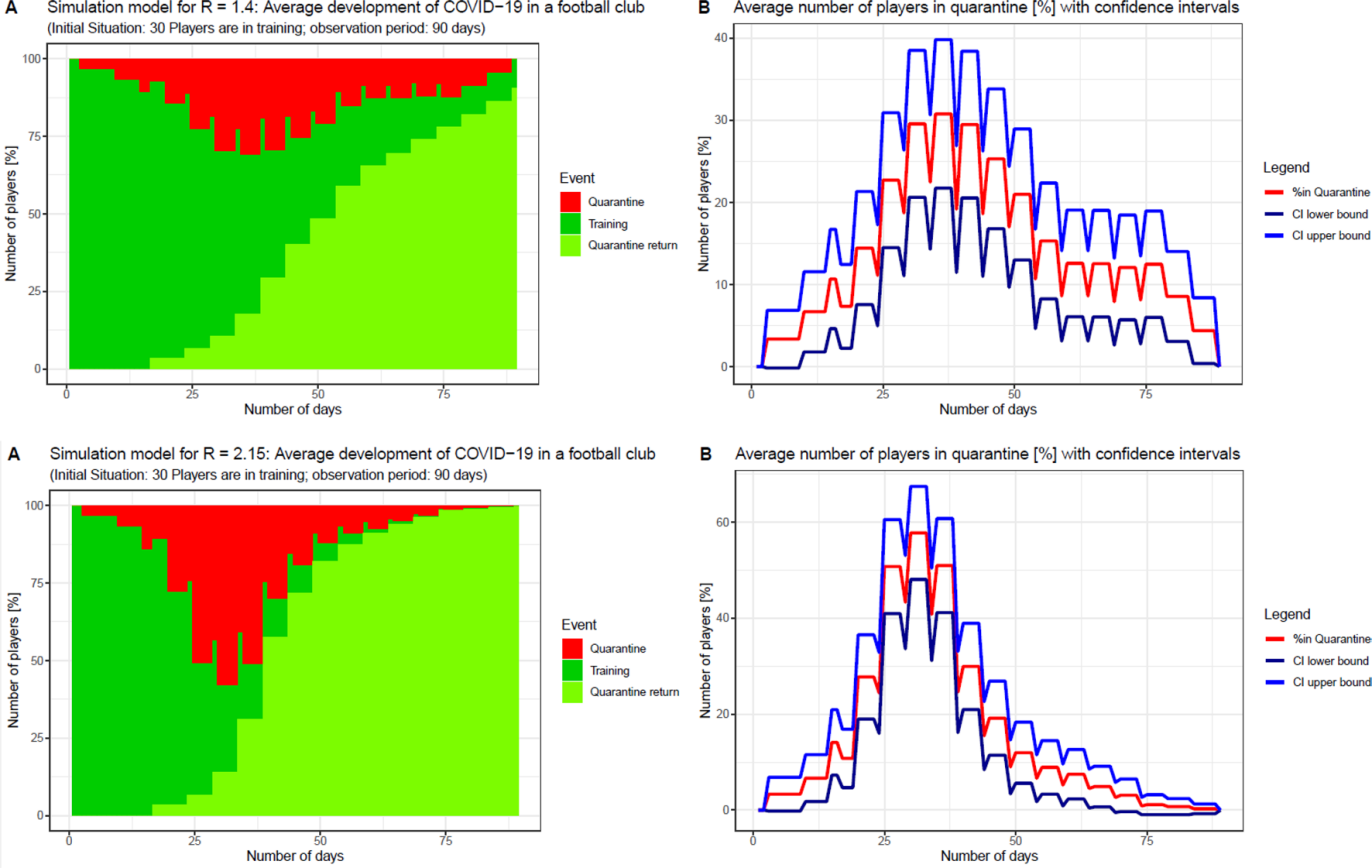
Simulation of hypothetical COVID-19 case among 30 players when R(0) = 1.4 or R(0) = 2.15.

Figure 3 illustrates the proportion of people infected with COVID-19 over a period of 90 days based on the assumptions listed in the method section. As can be seen, the proportion largely depends on the R(0) and differs substantially according to the actual number of infected players. In case of a higher R(0), multiple infections among players could eventually lead to a complete cessation of all training and competitive activities. For instance, in case of R(0) = 2.15, after approximately 25 days, about 50% of the player are in quarantine. After 90 days, all players have been infected and have returned from quarantine.

### Encountered implementation barriers

**Figure 4:**
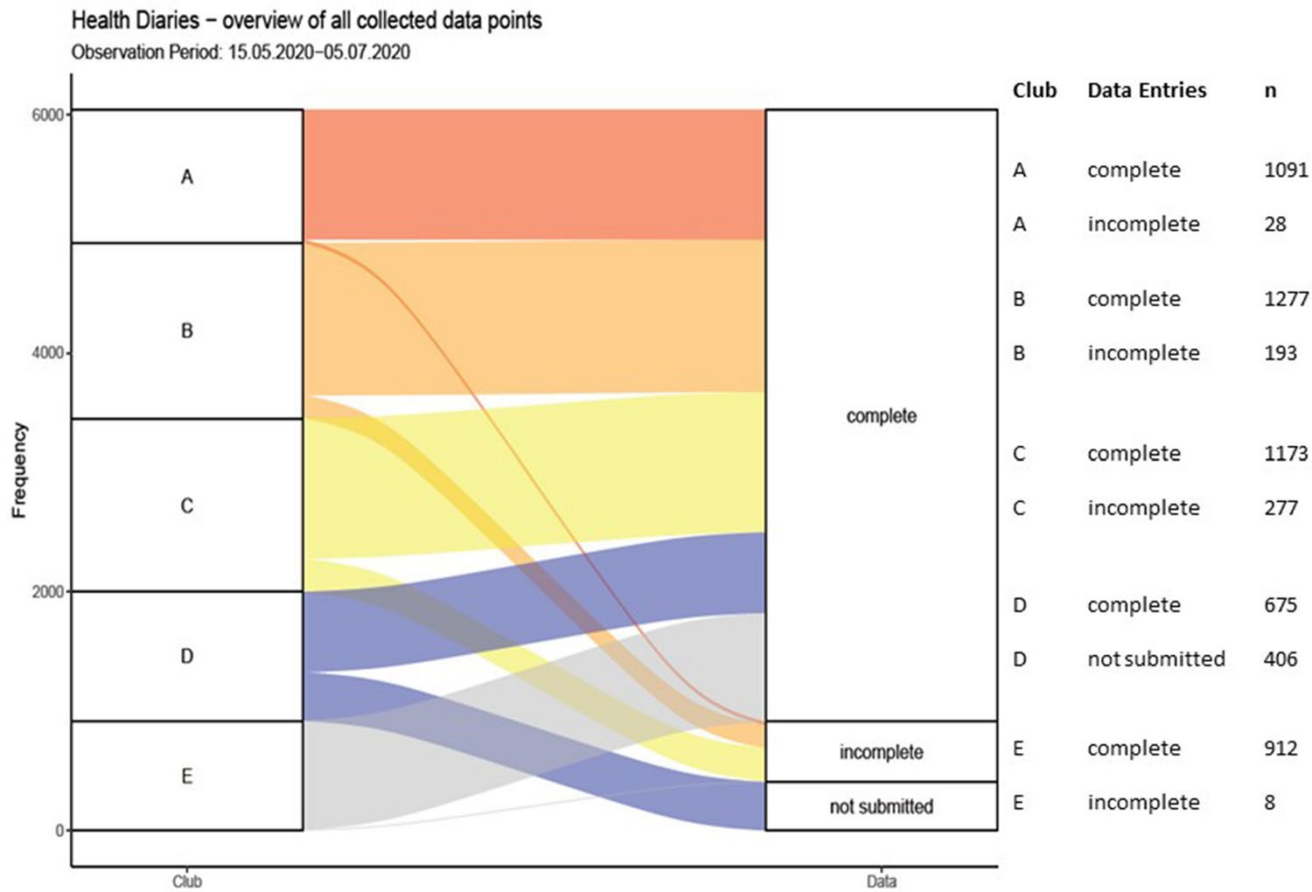
Completeness of health diaries from participating clubs during observation time.

Implementation barriers relating to health parameters were scarce. Data collection sheets were returned weekly to the researcher with the exception of one club, which failed to send information for a period of 2 weeks. Of the 146 players, 5305 data points were generated digitally and 329 data points were generated manually by the club-internal raters. Manually generated data points showed some weaknesses in terms of structuring of collected data. There were 5128 data points with complete information in all parameters (excluding oxygen saturation as obtaining uniform and valid devices was burdensome) and 506 data points with incomplete information. These data points were excluded from statistical analysis. Figure 4 summarizes the completeness of collected data in terms of generated data points.

**Figure 5:**
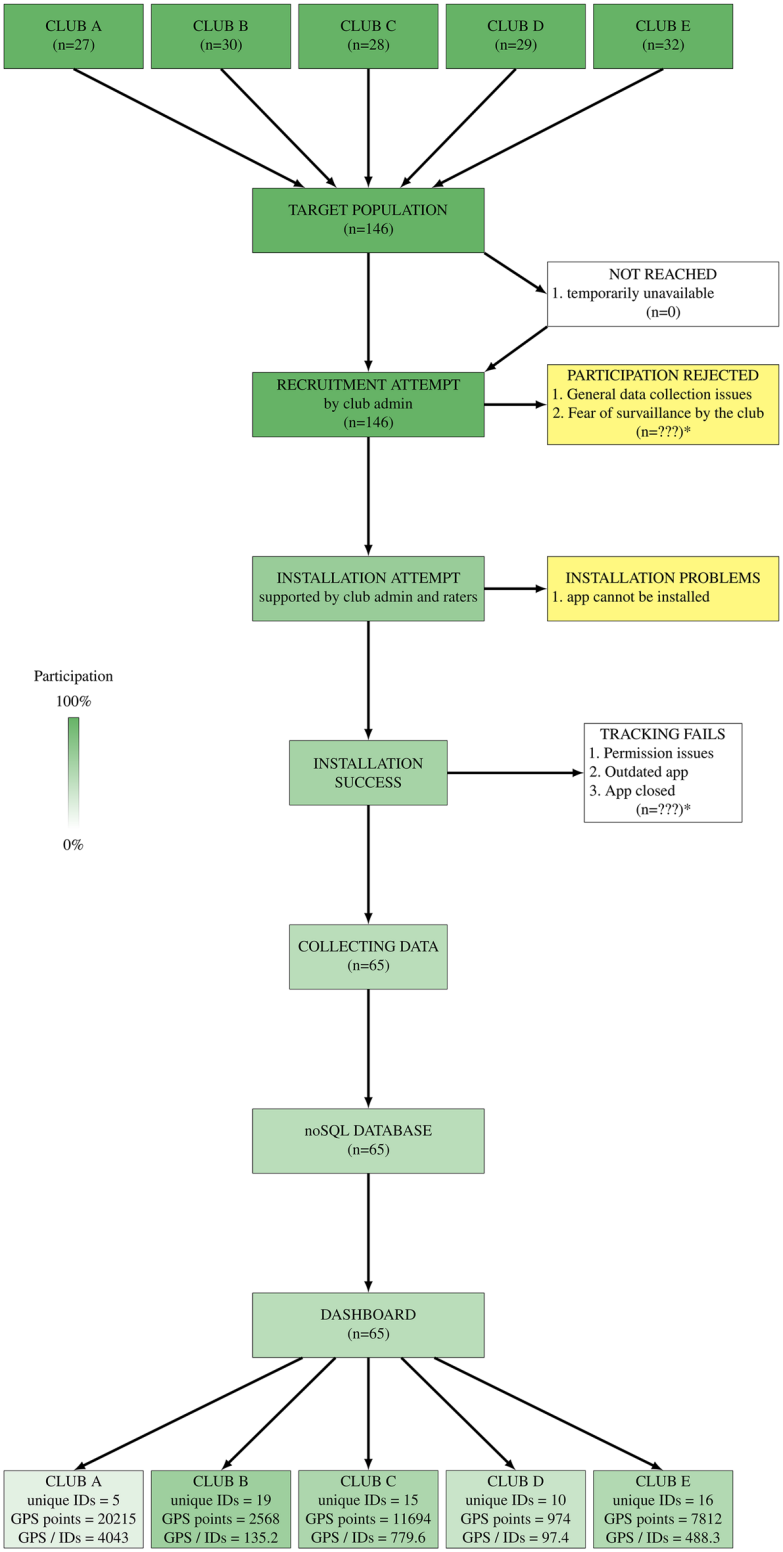
Flowchart of data collection through geo-tracking app and encountered barriers. *drop-out not assignable to specific reason, therefore n is unknown.

The implementation of the smartphone app for the collection of geo-tracking data was more challenging. Internal and external raters reported a reluctance of players to activate geo-tracking and a fear of their movements being monitored by their club. Additionally, technical challenges included unintended shutting down of the app through swiping, installation difficulties on certain smartphones and low density of collected data points occurred. The Flow-chart in Fig. 5 depicts the technically-related implementation barriers and their impact on the final sample with geo-tracking data available for analysis.

## Discussion

To our knowledge, this is one of the first papers to evaluate the process of safely resuming professional team contact sports during a pandemic. The results of our study suggest that even in times of a pandemic, the continuation of professional football is possible, on the condition of a rigorous surveillance concept. The ongoing pandemic is a challenge for sport clubs all over the world, who are under pressure to continue operating, while ensuring the highest possible level of safety. The quarantining of players can prevent clubs from putting their most important players on the pitch for crucial matches^11^.

Physical distancing and contact tracing combined with comprehensive COVID-19 vaccination are currently deemed the most effective strategies to reduce the risk of infection^12–15^. Regular PCR testing is also crucial for identifying infected persons early, although potential false negatives and the incubation period must be considered^16^. Even with meticulous adherence to all precautionary measures, transmission cannot be completely ruled out. The concept presented in the current study, along with the identification of implementation barriers, can inform future efforts to safely resume team contact sports.

During the observation period, none of the participants tested positive for COVID-19 and variations in health parameters were scarce; only sleep quality and level of recovery displayed slight variation. In line with previous studies reporting impaired sleep quality prior to competitions, these variations are likely attributable to tension and fatigue before and after games^17,18^. The weekly subjectively perceived risk of infection, which was included as a proxy for anxiety, significantly deviated from the weekly average when a COVID-19 case occurred in a manager in one of the clubs. This deviation was evident despite the fact that players were not in direct contact with the infected individual. This finding is particularly relevant when considering associations between anxiety and performance. Nieuwenhuys et al.^19^ suggested that anxiety can affect performance on various levels of operational control and potentially influence perceptual-motor behavior (including situational awareness and decision making). Accordingly, it seems plausible that player performance during competitions might be affected by the subjectively perceived risk of infection. This also highlights the importance of mental support strategies, including regular player education and reassurance, in addition to physical monitoring. Furthermore, adequate interdisciplinary communication (i.e. between management, trainers, physicians/physiotherapists and players) is paramount.

The absence of a COVID-19 case during the observation period of our study could be partially attributable to its low incidence and basic reproduction number in Austria during the observation period^2^. However, players may also travel to other, more affected countries and the situation in Austria is dynamic and evolving. Therefore, we simulated hypothetical scenarios in which the basic reproduction number of the virus rises. These simulations suggest that a worst-case scenario would result in discontinuation of training and competition. While these simulations are based on a number of assumptions, they highlight the necessity of comprehensive and ongoing player monitoring during the pandemic. Even in the case of asymptomatic infections, early detection is crucial to reduce the risk of further transmission and possible infection of vulnerable persons (e.g. relatives). Additionally, the number of players in professional squads is limited, meaning that a high R would end the league prematurely.

Continuous player monitoring is strenuous for data collectors and often an additional burden on top of daily duties. Efficient and user-friendly data collection is an important consideration; we provided digital options for data collection (i.e. a digital dashboard or excel sheets), training and on-site support in the early phase of the study. Despite this, some data entry errors were evident which naturally rendered data analyses more difficult. Accordingly, future monitoring concepts should focus on the training of data collectors to ensure consistent data collection.

The collection of geo-tracking data poses further challenges. Several countries have implemented digital contact tracing apps during the pandemic, mostly on a voluntary basis^20^. Geo-tracking mobile phone apps can potentially aid contact tracing, on the assumption of participants’ consent and compliance with applicable data protection regulations. These technical solutions are designed to enable faster identification and isolation of infected persons, but users’ concerns about privacy and governmental handling of personal data may hinder their application. It has been reported that the acceptance of app-based contact tracing is increased in persons who have comorbidities and always carry their phone^21^. Usually, young people like professional football players have greater technically affinity and should not encounter any difficulties in the installation of smartphone applications. However, in our study we encountered several barriers that hindered the use of the application. Firstly, there were several technical problems, secondly, the data collection appeared to be hindered by reluctance of players regarding geo-tracking, despite their informed consent to participate in the study. The reasons for this could not be established but may be attributable to data privacy concerns. Due to the strict data protection regulations in place and the anonymization of the data throughout the entire study, it was almost impossible to allocate precise numbers on each obstacle during the data collection process. It should be noted that participants of the study were reassured by our study assistants, their trainers, physicians and physiotherapists, that their data would be treated confidentially. The continuation of geo-tracking depends on the willingness of participants to collaborate. The inclusion of participants in the initial phase of monitoring prior to installation of the app may serve as a potential solution. In the context of large sporting events, not only the protection of players, but also the spectators needs to be considered and the use of apps is a potential solution^22^. Moreover, it should be emphasized that strict regulations only make sense if everyone adheres to them. This requires high motivation and cooperation of the whole team as they are also serving as role models for spectators.

Football is a popular sport in Europe, but the results of the study also hold relevance for to other team contact sports (e.g. rugby, basketball or American football) and settings (e.g. recreational and educational). Health parameters should be digitally recorded and closely monitored to enable quick responses. Furthermore, mental health aspects should be included as part of a comprehensive monitoring concept. Mobile phone contact tracing apps can potentially support the identification and containment of infections. The results of this study can inform the development of future prevention and monitoring strategies in professional football and beyond, potentially serving as a blueprint for the safe continuation of sports and physical activity, across a broad range of settings, during and following a pandemic such as COVID-19.

## Data Availability

The datasets and codes generated during and/or analysed during the current study are available from the corresponding author on reasonable request.
